# 3D Transgastric Views in Transesophageal Echocardiography (TEE) to Diagnose Isolated Pulmonary Valve Endocarditis: When the Standard Views Fall Short

**DOI:** 10.7759/cureus.103524

**Published:** 2026-02-13

**Authors:** Alex P Rodriguez, Sonia I Vicenty-Rivera

**Affiliations:** 1 Cardiology, Bruce W. Carter Veterans Affairs (VA) Miami Healthcare System, Miami, USA; 2 Research and Development, Veterans Affairs Caribbean Healthcare System, San Juan, PRI

**Keywords:** cardiac magnetic resonance, pulmonary vein endocarditis, right-sided infective endocarditis, transesophageal echocardiography, transthoracic echocardiography

## Abstract

Isolated pulmonary valve endocarditis (PVE) is a rare and diagnostically challenging form of right-sided infective endocarditis, often lacking the typical findings seen with left-sided or tricuspid valve involvement. We describe an 83-year-old man with end-stage renal disease on hemodialysis, who developed persistent *Staphylococcus aureus* bacteremia despite appropriate antibiotics and catheter exchange. Standard diagnostic methods, including physical examination, laboratory tests, and transthoracic echocardiography, were unremarkable. Initial transesophageal echocardiography (TEE) with conventional views was limited by acoustic shadowing. Ultimately, transgastric two-dimensional (2D) and three-dimensional (3D) TEE imaging revealed vegetation on the pulmonary valve, leading to the diagnosis. The patient improved with ongoing therapy, and further imaging was deferred by agreement. This case highlights the need to consider isolated PVE in patients with persistent bacteremia and intravascular devices, even without classic risk factors. It also emphasizes the value of advanced imaging, particularly transgastric 3D TEE, for detailed assessment of the pulmonary valve when conventional studies are inconclusive. A multidisciplinary approach involving infectious diseases, nephrology, and cardiology is essential for prompt diagnosis and effective management. In summary, comprehensive imaging and a high index of suspicion are critical for identifying isolated PVE, which should remain a differential diagnosis in select high-risk patients with unexplained bacteremia.

## Introduction

Right-sided infective endocarditis (IE) is a relatively uncommon clinical entity, representing approximately 1-10% of all IE cases, depending on the literature cited [1,2]. Isolated involvement of the pulmonary valve (PV) is exceedingly rare, with pulmonary valve endocarditis (PVE) comprising less than 2% of all reported IE cases [3,4]. It is often misdiagnosed and requires heightened clinical suspicion due to the patient's nonspecific respiratory symptoms. Among the differential diagnoses of this condition are infectious, thrombotic, neoplastic, and inflammatory etiologies. The most common mimics are pulmonary embolism and pulmonary artery sarcoma, both of which can present with fever, dyspnea, and pulmonary artery filling defects on CT angiography. The majority of PVE cases are associated with established risk factors, including intravenous drug use, congenital heart disease, or immunocompromised conditions [3]. Right-sided IE generally has a better prognosis than left-sided IE, with lower mortality rates [1-3]. However, delays in diagnosis and treatment, as well as complications, such as septic pulmonary emboli and valvular destruction, significantly increase morbidity and mortality.

We present a clinically significant case of isolated PVE in a patient with end-stage renal disease (ESRD) undergoing hemodialysis via an indwelling central venous catheter. The patient developed persistent methicillin-sensitive *Staphylococcus aureus* (MSSA) bacteremia, a scenario with substantial implications for morbidity and mortality in this population. Initial transthoracic echocardiography (TTE) and adjunct imaging failed to reveal significant valvular dysfunction or vegetations. Despite prompt catheter exchange and targeted antimicrobial therapy, bacteremia persisted. Subsequent transesophageal echocardiography (TEE) was pivotal in identifying a vegetation localized to the anterior leaflet of the PV, with no evidence of involvement of other cardiac valves or intracardiac structures. TEE further corroborated the presence of trivial-to-mild pulmonary regurgitation, consistent with prior TTE findings. This case underscores the importance of maintaining a high index of suspicion for PVE in at-risk populations and highlights the diagnostic value of TEE when initial studies are inconclusive.

## Case presentation

An 83-year-old gentleman with a history of chronic kidney disease, currently requiring hemodialysis (HD) via a central venous catheter, presented to the emergency department with complaints of weakness and diarrhea. Additional past medical history was notable for coronary artery disease, cerebrovascular accident, and diabetes. In the emergency department, he appeared hemodynamically and clinically stable, with a blood pressure of 145/90 mmHg, a heart rate of 90 beats per minute (bpm), and an oxygen saturation of 97%; however, his white blood cell count was elevated at 14,000×10^9^/L.

Upon admission, the patient became febrile, and blood cultures were drawn, which grew methicillin-sensitive *Staphylococcus aureus* (MSSA). Following recommendations from the infectious disease team, the HD catheter was exchanged, and the patient was initiated on renally adjusted dosing of vancomycin. Transthoracic echocardiography (TTE) demonstrated a left ventricular ejection fraction of 60-65% with no significant valvular regurgitation. The pulmonary valve was suboptimally visualized, and no clear abnormalities were appreciated. Both the right atrium and right ventricle were normal in size, with the right ventricle demonstrating normal systolic function. There were no clinical signs of heart failure, conduction abnormalities, or embolic phenomena observed.

Despite appropriate antibiotic therapy following catheter exchange, the patient’s blood cultures remained persistently positive for MSSA, and no definitive source of infection was identified. The infectious disease consultants recommended transesophageal echocardiography (TEE) to facilitate a more detailed evaluation for possible intracardiac infection.

**Figure 1:**
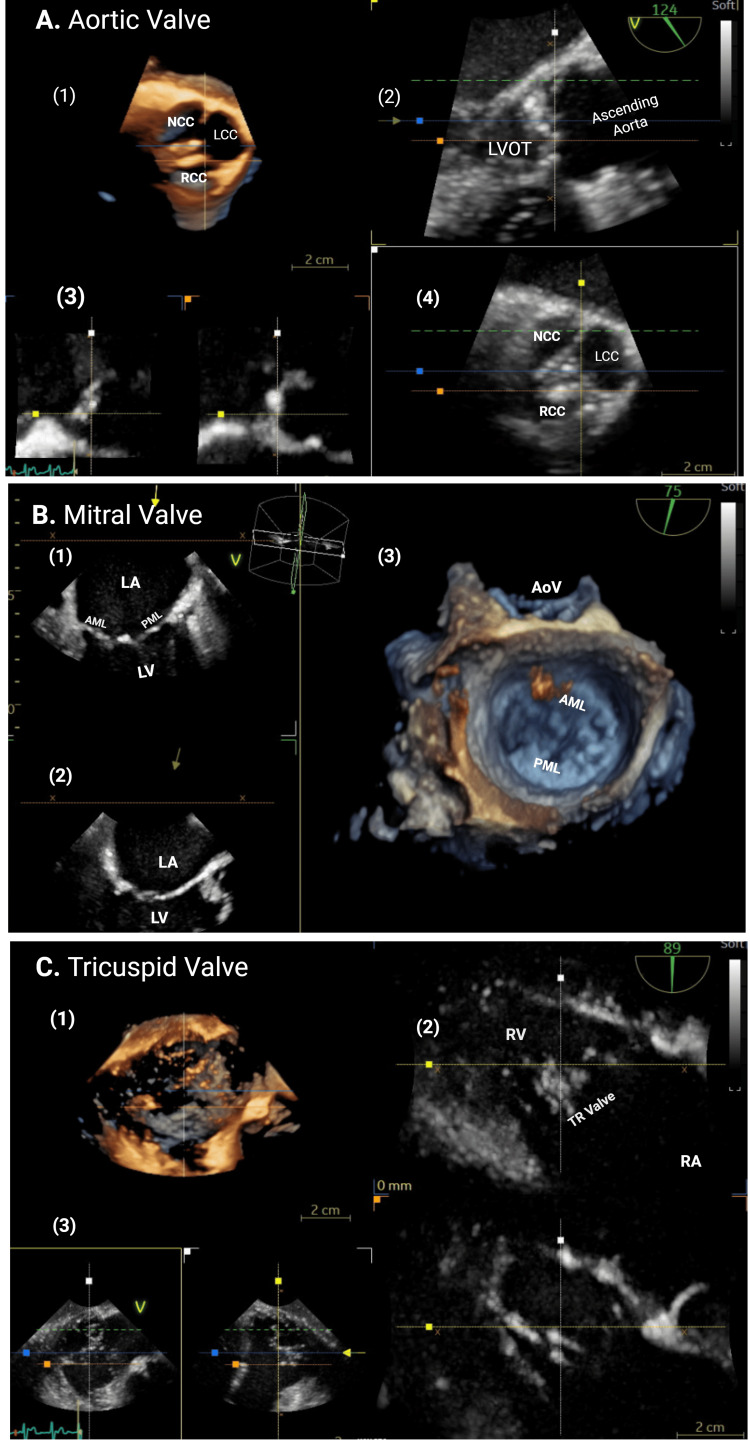
Normal aortic, mitral, and tricuspid valve anatomy demonstrated by 2D and 3D echocardiography without infective endocarditis. Panel A illustrates the aortic valve with various two‑dimensional views ({A.2} PLAX view, {A.3} biplane view, and {A.4} basal SAX view) and three‑dimensional transthoracic echocardiographic view (A.1). The NCC, LCC, and RCC are visualized along with the LVOT and ascending aorta. The 3D en-face view demonstrates trileaflet morphology and cusp coaptation. Panel B illustrates imaging of the mitral valve and left‑sided chambers (B.1 and B.2). The AML, PML, LA, and LV are shown in orthogonal 2D planes. The 3D reconstruction highlights leaflet curvature, annular geometry, and spatial relationship to the aortic valve (B.3). Panel C illustrates two‑dimensional (C2-biplane RV inflow views and C3-biplane view) and three‑dimensional views of the tricuspid valve and right‑sided chambers (C.1). The RA, RV, and tricuspid valve leaflets are displayed in longitudinal and en‑face perspectives, allowing assessment of leaflet motion and annular structure. NCC: noncoronary cusp; LCC: left coronary cusp; RCC: right coronary cusp; LVOT: left ventricular outflow tract; AML: anterior mitral leaflet; PML: posterior mitral leaflet; LA: left atrium; LV: left ventricle; RA: right atrium; RV: right ventricle; PLAX: parasternal long axis; SAX: short axis

TEE was performed under monitored anesthesia care and was well tolerated. It demonstrated normal biventricular systolic function. The aortic, mitral, and tricuspid valves appeared anatomically normal and without significant pathology (Figure 1 panels A-C). Detailed imaging of the sinuses of Valsalva, left ventricular outflow tract, and aortomitral intervalvular fibrosa revealed no abnormalities (Figure 1 panels A-C). Additionally, there was no evidence of paravalvular extension, abscess, intracardiac fistula, or intracardiac shunt.

**Figure 2:**
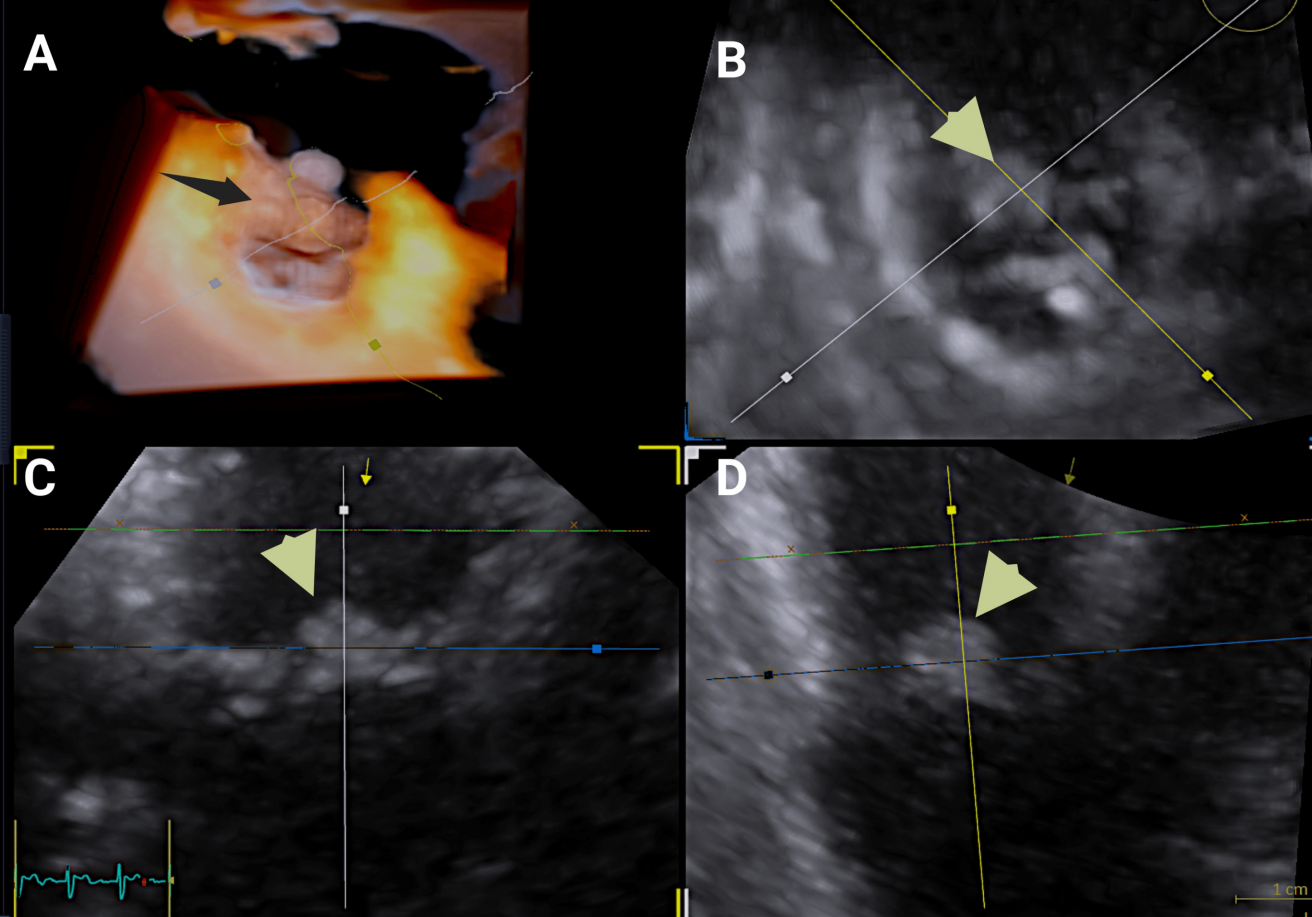
Flexislice transillumination 2D and 3D transesophageal echocardiography images. Flexislice transillumination 2D and 3D transesophageal echocardiography of the pulmonary valve (panel A). A vegetation was identified attached to the anterior cusp of the pulmonary valve (black and yellow/green arrows) (panels B-D).

Evaluation of the pulmonary valve by TEE proved technically challenging. Both the high esophageal view (0° multiplane angle) and the mid-esophageal right ventricular inflow-outflow view (typically 45-70°) were limited by acoustic shadowing. However, transgastric views permitted optimal visualization of the pulmonary valve (Video 1, Figure 2 panels A-D).

**Video 1:** Two-dimensional-transgastric multiplane views at 0° and 90° of the pulmonary valve showing an oscillating mass-like structure (vegetation) on the surface of the anterior pulmonary valve cusp. There are no echolucent areas around the pulmonary valve, excluding abscess formation.

Three-dimensional (3D) and Doppler imaging were employed for further assessment, and multiplanar reconstruction (MPR) enabled detailed evaluation of the valve leaflets and perivalvular space (Video 2). These studies confirmed the presence of vegetation on the anterior leaflet of the pulmonary valve, with no involvement of other cardiac structures. Trivial-to-mild pulmonary regurgitation was also noted.

**Video 2:** Two-dimensional and three-dimensional transesophageal views of the pulmonary valve and RVOT, shown in original resolution. There is an oscillating mass-like structure attached to the endocardial surface of the pulmonary valve anterior cusp. RVOT: right ventricular outflow tract

Antibiotic therapy was continued. Chest computed tomography (CT) was performed and excluded embolic complications. The patient remained hemodynamically and clinically stable, and his hospital course was uncomplicated.

## Discussion

**Figure 3:**
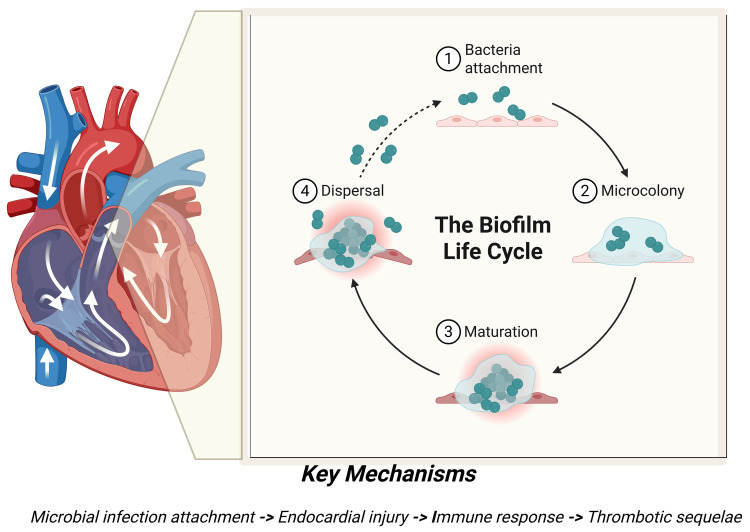
This figure outlines the biofilm life cycle associated with microbial infections. This figure illustrates the key stages as follows: attachment of bacteria, maturation of biofilms, microcolony development, and dispersal. The accompanying diagram highlights the relevance of these mechanisms to endocardial injury, immune response, and thrombotic outcomes, providing insight into the pathogenesis of biofilm-related infections. This image is created by the author (Sonia I. Vicenty-Rivera) of this study.

Pulmonary valve endocarditis (PVE) is an exceptionally rare clinical entity, typically described in case reports and small case series owing to its low prevalence (Figure 3)*. *The rarity of this condition is multifactorial, largely attributable to physiological and anatomical factors. These include lower pressures within the right heart, reduced oxygen content in venous blood, lower jet velocities, decreased wall stress, and a distinct endothelial lining and vascularization of the pulmonary valve [3-5]. In cases of PVE, vegetations are predominantly located on the ventricular aspect of the valve.

Intravenous drug use remains the most frequently reported predisposing factor; however, several other important risk factors have been identified. These include the presence of central venous catheters, a history of alcohol abuse, recent dental procedures, orthotopic organ transplantation, congenital heart disease, and states of immunosuppression [3-7]. The most common pathogens are *Staphylococcus aureus *(particularly in intravenous drug users), Enterococcus species, and Streptococcus species. However, Gram-negative bacteria and fungi (e.g., Candida* *species) are rarely observed.

Right-sided infective endocarditis (IE), including PVE, often presents with pulmonary symptoms, such as cough, pleuritic chest pain, or hemoptysis. A diastolic murmur of pulmonary insufficiency may be appreciated on physical examination, though this is typically a late finding. Transthoracic echocardiography (TTE), despite its known limitations in visualizing the pulmonary valve, has been reported to detect abnormalities in up to 91% of cases [3].

The present case represents an atypical presentation in several respects. The patient exhibited none of the classic pulmonary symptoms, and TTE failed to suggest pulmonary valve involvement. This underscores the diagnostic challenges associated with isolated PVE and highlights the need for a high index of suspicion in patients with persistent bacteremia, even in the absence of clinical or imaging findings on initial evaluation [8]. Notably, *Staphylococcus aureus* remains the most commonly implicated pathogen in PVE, consistent with our findings [7].

Transesophageal echocardiography (TEE) remains a class I recommendation in multiple clinical scenarios for evaluating infective endocarditis, owing to its superior temporal and spatial resolution. However, in cases of pulmonary valve endocarditis, TEE presents unique challenges [1]. The pulmonary valve is the most anterosuperior cardiac structure and, consequently, the farthest from the TEE probe, often resulting in suboptimal imaging. In this case, both high and mid-esophageal views failed to provide diagnostic-quality images or adequate Doppler interrogation due to significant acoustic shadowing. In contrast, transgastric views, though technically demanding, allowed for high-quality two-dimensional (2D) and three-dimensional (3D) imaging of the pulmonary valve, including comprehensive Doppler assessment. These views were critical in identifying the vegetation and confirming the absence of additional valvular or perivalvular involvement. This underscores that anatomical factors can limit image quality in certain planes, and a thorough evaluation requires adaptation to such challenges to ensure higher diagnostic confidence.

With advances in multimodality imaging, newer protocols now allow for comprehensive assessment in the diagnosis of infective endocarditis (IE), monitoring of treatment response, and detection of complications [9,10]. Cardiac computed tomography (cCT) has emerged as a valuable adjunct, particularly when transthoracic echocardiography (TTE) and transesophageal echocardiography (TEE) are inconclusive despite high clinical suspicion. In addition to anatomical visualization, cCT provides high sensitivity for detecting paravalvular extension of infection and periannular complications. In a study by Habets et al., the use of cCT resulted in major diagnostic and therapeutic changes in 21% of patients evaluated [11]. Several advantages of cCT include excellent spatial resolution and short acquisition times. Additionally, with appropriate heart rate and arrhythmia management, precise anatomic evaluation of the right ventricular outflow tract, pulmonary artery, and epicardial vessels is feasible. The main limitations of cCT are exposure to ionizing radiation and the use of iodinated contrast agents, which may be problematic in patients with pre-existing renal dysfunction. In such cases, close collaboration with nephrology can help mitigate the risk of contrast-induced nephropathy through pre-procedure optimization and post-procedure monitoring.

Cardiac magnetic resonance (CMR) is a valuable modality for the anatomical evaluation of the pulmonary valve (PV) and for the accurate quantification of pulmonary regurgitation [10]. Pulmonary insufficiency typically appears on CMR as a diastolic, broad, dark jet caused by signal dephasing from turbulent flow. Quantitative assessment can be achieved using through-plane phase-contrast imaging at the level of the main and branch pulmonary arteries, enabling identification and measurement of holodiastolic flow reversal. In the absence of other valvular lesions, significant discrepancies in left and right ventricular stroke volumes can serve as an indirect method to estimate pulmonary regurgitant fraction. Furthermore, CMR allows for precise measurement of right ventricular volumes and ejection fraction using steady-state free precession (SSFP) or gradient-recalled echo (GRE) cine sequences. These sequences are typically both respiratory- and ECG-gated, which may present challenges for certain patients. For such individuals, free-breathing techniques are available, and newer compressed-sensing sequences can be used, albeit with a trade-off in spatial resolution. Emerging techniques, such as four-dimensional (4D) flow imaging, provide comprehensive, time-resolved, phase-contrast evaluation of blood flow across a three-dimensional vascular volume. This modality facilitates advanced flow visualization and quantification, but remains limited by prolonged acquisition times and the potential for patient fatigue during scanning.

In addition to cardiac computed tomography (cCT) and cardiac magnetic resonance (CMR), nuclear imaging modalities have emerged as valuable adjuncts in the evaluation of infective endocarditis (IE), particularly in diagnostically challenging cases. Fluorodeoxyglucose positron emission tomography (FDG-PET) has shown promise in this context. In a meta-analysis of 13 studies involving 537 patients, Mahmood et al. demonstrated that FDG-PET significantly improves diagnostic accuracy for prosthetic valve endocarditis (PVE), with a reported sensitivity of 80.5% and specificity of 73.1% compared to the modified Duke criteria [12]. Although less extensively studied, radiolabeled leukocyte scintigraphy also shows potential as an additional imaging tool in the diagnosis of IE, particularly in complex or equivocal cases. Its ability to localize active infection may complement other modalities when standard echocardiographic or radiographic imaging is inconclusive.

This case is consistent with existing literature regarding predisposing factors for isolated pulmonary valve endocarditis. In our patient, the presence of an indwelling hemodialysis catheter, along with probable immunocompromise due to diabetes, end-stage renal disease, advanced age, and multiple comorbidities, likely contributed to increased susceptibility. We assert that every case of suspected endocarditis warrants a thorough and comprehensive evaluation, regardless of initial clinical or imaging findings. In our case, no other valves or intracardiac structures were involved. While standard transesophageal echocardiographic views were nondiagnostic due to significant acoustic shadowing, transgastric imaging, including both three-dimensional and Doppler interrogation, enabled definitive assessment of the pulmonary valve [13]. The inherent difficulty in visualizing this most anterior cardiac structure, combined with patient-related limitations, may partly explain the wide variability in the reported prevalence of isolated PVE in the literature.

## Conclusions

This case highlights the rarity and diagnostic challenges of isolated pulmonary valve endocarditis (PVE), especially when typical clinical features are absent. The patient lacked classic pulmonary symptoms and physical findings, and initial transthoracic echocardiography (TTE) did not detect valvular involvement. Early diagnosis is often delayed due to nonspecific symptoms and the limited sensitivity of TTE. The patient’s risk factors, including an indwelling hemodialysis catheter, diabetes, end-stage renal disease, advanced age, and multiple comorbidities, likely contributed to PVE development. Given the low incidence and nonspecific presentation of PVE, this case underscores the need for a multidisciplinary diagnostic approach and advanced imaging. Transesophageal echocardiography (TEE), computed tomography (CT), and positron emission tomography/computed tomography (PET/CT) are often necessary. When standard TEE views were limited by acoustic shadowing, transgastric views allowed for comprehensive two- and three-dimensional, as well as Doppler, assessment of the pulmonary valve. No additional intracardiac involvement was found, consistent with isolated PVE. Anatomical challenges and patient-specific factors may contribute to variability in reported prevalence. In summary, this case is unique because it illustrates how isolated pulmonary valve endocarditis can present without classic symptoms, evade initial detection with routine imaging, and require advanced, patient-specific techniques to establish the diagnosis. The combination of unusual clinical presentation, absence of additional intracardiac involvement, and diagnostic reliance on innovative echocardiographic approaches distinguishes this case from typical endocarditis scenarios. This underscores the necessity of high clinical vigilance and adaptability in diagnostic strategies when confronted with rare cardiac infections.
